# Preclinical Investigation of [^212^Pb]Pb-DOTAM-GRPR1 for Peptide Receptor Radionuclide Therapy in a Prostate Tumor Model

**DOI:** 10.2967/jnumed.124.268101

**Published:** 2024-11

**Authors:** Amal Saidi, Tania A. Stallons, Amy G. Wong, Julien J. Torgue

**Affiliations:** 1 Orano Med SAS, Paris, France; and; 2 Orano Med LLC, Plano, Texas

**Keywords:** [^212^Pb]Pb-DOTAM-GRPR1, cancer, peptide, targeted α-therapy

## Abstract

The role of gastrin-releasing peptide receptor (GRPR) in various diseases, including cancer, has been extensively studied and has emerged as a promising therapeutic target. In this study, we successfully achieved the use of [^212^Pb]Pb-DOTAM-GRPR1, comprising the α-particle generator, ^212^Pb, combined with a GRPR-targeting peptide, GRPR1, in a prostate cancer model. **Methods:** Pharmacokinetics, toxicity, radiation dosimetry, and efficacy were assessed in GRPR-positive prostate tumor–bearing mice after intravenous administration of [^212^Pb]Pb-DOTAM-GRPR1 (where DOTAM is 1,4,7,10-tetrakis(carbamoylmethyl)-1,4,7,10-tetraazacyclododecane). **Results:** Preclinical studies have shown tumor targeting of up to 5 percent injected dose per gram over 24 h, and optimization of the drug formulation and quantity has led to minimized oxidation and off-target binding, respectively. Particularly, an increase in peptide amount from 28 to 280 ng was shown to reduce off-target uptake, especially at the level of the pancreas, by about 30%. Furthermore, dosimetry studies confirmed the kidney as the dose-limiting organ, and toxicity studies revealed that a nontoxic dose of up to 1,665 kBq could be injected into mice. Efficacy studies indicated a median survival time of 9 wk in the control group, which received only a buffer solution, compared with 19 wk in the group that received 4 injections of 370 kBq at 3-wk intervals. **Conclusion:** Taken together, these combined data demonstrate the safety, tolerability, and efficacy of [^212^Pb]Pb-DOTAM-GRPR1, thus warranting further exploration in clinical trials.

## Visual Abstract

**Figure:**
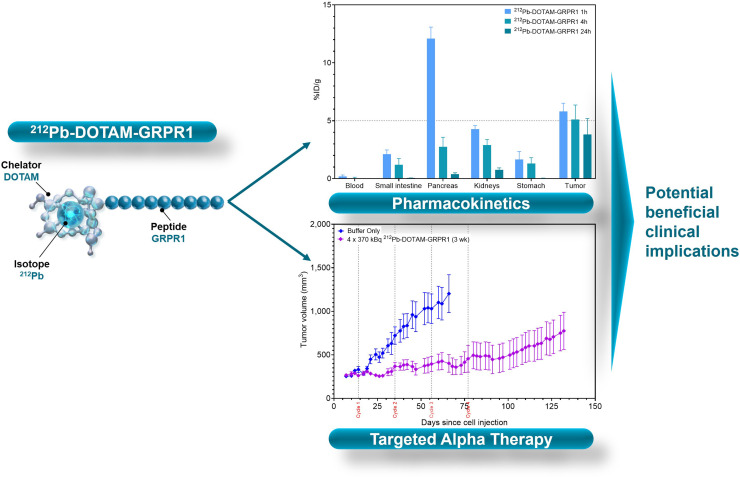

Targeted α-therapy is an emerging radiopharmaceutical therapy that selectively delivers α-particle radiation to cancer cells. α-particles offer several advantages over β-emitters, such as a shorter pathlength, which theoretically limits radiation exposure to nontargeted tissues, and the ability to induce cell death with fewer particles because of a higher linear energy transfer, which greatly increases the likelihood of irreparable double-stranded DNA breaks that are fatal to the cell (1). α-particle–emitting radionuclides are delivered to tumors by chelation to monoclonal antibodies, peptides, or small molecules that recognize tumor-associated antigens or cell surface receptors (2).

^212^Pb is an α-particle–emitting isotope that has a half-life of 10.6 h and is well suited for targeted α-therapy because of its unique physical properties (3). In preclinical studies, the efficacy of ^212^Pb as an antitumor agent has been demonstrated in various animal cancer models, including peritoneal cancer (4), prostate cancer (5), melanoma (6), pancreatic cancer (7), breast cancer (8), neuroendocrine tumors (9), and hematologic cancers such as multiple myeloma (10), lymphocytic leukemia, and non-Hodgkin lymphoma (11). Similarly, several clinical trials have investigated the safety and efficacy of ^212^Pb-based targeted α-therapy and showed promising antitumor activity (12,13).

Gastrin-releasing peptide receptor (GRPR) is a G-protein–coupled receptor of the bombesin receptor family. It is overexpressed in many tumor types, including prostate (14–16), breast (17), lung (18), colorectal (19), and cervical (20) cancers and cutaneous melanoma (21). Overexpression of GRPR was found in 63%–100% of primary prostate cancers and more than 50% of lymph node and bone metastases. In breast cancer, Morgat et al. (17) reported that GRPR overexpression was found in 75.8% of all tumors analyzed and was strongly associated with estrogen receptor positivity.

Given the variety of malignancies that overexpress GRPR, numerous radiopharmaceuticals have been developed to target GRPR-positive tumors. Studies have shown that GRPR antagonists have superior properties to those of GRPR agonists, including higher tumor uptake and lower accumulation in physiologic GRPR-expressing healthy tissues (14,16,22). Therefore, we developed the radiopharmaceutical [^212^Pb]Pb-DOTAM-GRPR1, comprising the α-emitter ^212^Pb radioisotope, the metal chelator DOTAM (1,4,7,10-tetrakis(carbamoylmethyl)-1,4,7,10-tetraazacyclododecane), and a GRPR-targeting antagonist, GRPR1. On binding to GRPR, as ^212^Pb decays, it emits an α-particle. The short range of α-particles (50–100 μm) ensures that they deposit energy primarily within the tumor cell while sparing surrounding healthy tissues. The high linear energy transfer of α-particles leads to DNA double-strand breaks in the tumor cell. These breaks are difficult for the cell to repair, resulting in irreversible damage. Tumor cells would then undergo apoptosis or necrosis because of this extensive DNA damage. Adjacent tumor cells that do not directly bind to [^212^Pb]Pb-DOTAM-GRPR1 can also be affected. In this article, several preclinical studies using relevant xenograft models of GRPR-overexpressing prostatic tumors have demonstrated the benefits of [^212^Pb]Pb-DOTAM-GRPR1. These data supported the initiation of a phase I clinical trial (23).

## MATERIALS AND METHODS

### Cell Line and Mice

The PC-3 prostate cancer cell line was purchased from ATCC. Cells were maintained in F12K medium (Gibco) containing 10% fetal bovine serum (Gibco). Athymic nude and CD-1 mice were purchased from Envigo. All experiments were conducted using male mice unless otherwise stated. Animal studies were approved by the Institutional Animal Care and Use Committee and performed in compliance with guidelines from the Public Health Service Policy and Animal Welfare Act of the United States.

### Manufacturing and Radiolabeling

**FIGURE 1.**
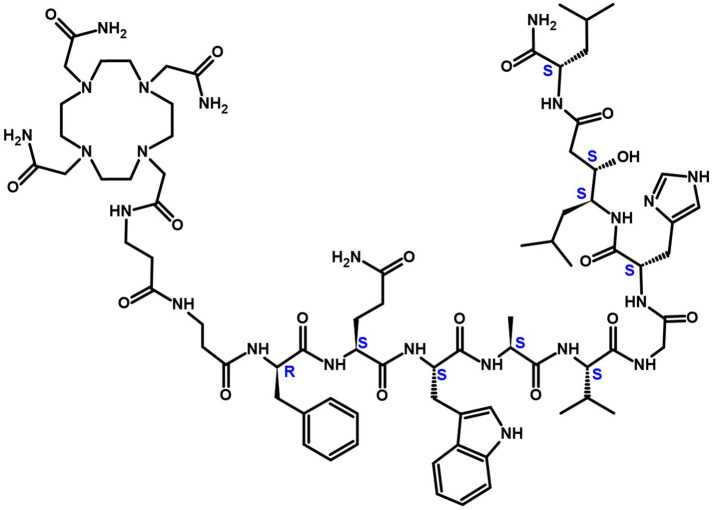
DOTAM-GRPR1 chemical structure. DOTAM-GRPR1 is comprised of DOTAM chelator linked to 2-amino-acid linker (β-Ala-β-Ala) followed by 9-amino-acid peptide GRPR (dPhe-Gln-Trp-Ala-Val-Gly-His-Sta-Leu-NH_2_) during solid-phase peptide synthesis.

DOTAM-GRPR1 (C_77_H_119_N_23_O_17_, Fig. 1) was manufactured by Macrocyclics using Fmoc solid-phase peptide synthesis. The peptide was isolated via reverse-phase high-performance liquid chromatography. Chelation was achieved by adding ^212^Pb in ammonium acetate (Sigma-Aldrich) to DOTAM-GRPR1 alone or in the presence of 5% ethanol (Spectrum Chemical), up to 30 mM ascorbic acid (Honeywell), and 0.02% polysorbate 80 (J.T. Baker). Experiments were conducted for 10 min at either room temperature or 50°C.

Instant thin-layer chromatography was used to confirm chelation of more than 95%. Before injection, samples were diluted in either 5% ethanol, 20 mM ascorbic acid, 0.02% polysorbate 80, 60 mM ammonium acetate, or 0.08% polysorbate 20 (Fisher Scientific) in 0.9% sodium chloride (Hospira) to obtain the desired activity per injection.

### Cell-Binding Assay

The dissociation constant (K_d_) was calculated in GRPR-expressing PC-3 cells by growing 35,000 cells in 96-well plates for 24 h. Concentrations of 0.5–64 nM [^212^Pb]Pb-DOTAM-GRPR1 were incubated with PC-3 cells for 10 min at 37°C. A second set of samples containing 0.5–64 nM [^212^Pb]Pb-DOTAM-GRPR1 with a 10 μM excess of cold DOTAM-GRPR1 were also examined to determine nonspecific binding. Experiments were performed in triplicate for each concentration. Cells were then washed with phosphate-buffered saline, and the presence of radioactivity was measured in the cells from each well. Binding curves were generated by subtracting nonspecific binding from total binding, and the K_d_ was calculated using GraphPad Prism software.

### Tumor Models

For all tumor studies, 1 × 10^6^ PC-3 cells were implanted subcutaneously into the right flank of each mouse in a mixture of equal volumes of HC-Matrigel (high concentration; Corning) and RPMI medium (Gibco) and grown to a volume of about 200–300 mm^3^.

### Biodistribution Studies

PC-3 tumors were grown in male mice until an approximate tumor volume of 300 mm^3^ was reached. For all doses, 100 μL of [^212^Pb]Pb-DOTAM-GRPR1 were administered to mice via the tail vein, and the mice were euthanized at predetermined time points. The background was automatically subtracted from the counts. A standard was also used for decay correction, and the percent injected dose (%ID) per gram of tissue was calculated for each organ collected.

### Dosimetry Studies

A biodistribution with 5 time points was used to calculate the absorbed radiation doses from [^212^Pb]Pb-DOTAM-GRPR1, performed by Radiopharmaceutical Imaging and Dosimetry, LLC (Rapid). Physical decay was used to extrapolate after the last time point. The human equivalent time-integrated activity coefficient for both sexes was calculated by extrapolating mouse biodistribution data to human using standard methods assuming that tissue concentration divided by whole-body concentration in mice is the same as that in humans.

### α-Imaging

Ex vivo assessment of [^212^Pb]Pb-DOTAM-GRPR1 localization and microdistribution was performed on frozen sections (10–12 μm) of PC-3 xenograft tumors placed on a phosphor sheet (Eljen Technology) and imaged using a high-sensitivity iXon Ultra 888 EMCCD camera (Andor). Images were analyzed using Andor Solis software (ImageJ).

### Radio–High-Performance Liquid Chromatography Analysis

[^212^Pb]Pb-DOTAM-GRPR1 was analyzed on an Agilent 1220 high-performance liquid chromatograph using a C18 reverse-phase column (Restek) with an acetonitrile gradient. Fractions were collected from the column every 6–10 s for up to 10 min and then analyzed for radiometric detection using an automatic γ-counter (Perkin Elmer Wizard^2^ 2470).

### Toxicity Studies

Male athymic nude mice intravenously received either 1 injection of 370, 555, 740, 1,110, or 1,665 kBq or, at 2-wk intervals, 3 injections of 370 or 555 kBq of [^212^Pb]Pb-DOTAM-GRPR1. Control mice received buffer only. Animals were weighed 3 times per week and monitored daily for signs of pain or distress over a 4-wk period after the last injection. Blood was sampled via the submandibular vein using potassium–ethylenediaminetetraacetic acid capillaries and tubes (Greiner Bio-One), and complete cell blood counts were performed using a VETSCAN HM5 hematology analyzer (Abaxis/Zoetis). Animals were euthanized at the scheduled termination or when the termination criteria were met.

### Efficacy Study

PC-3 tumor–bearing animals were injected with 3 or 4 administrations of 100 μL of [^212^Pb]Pb-DOTAM-GRPR1 or negative control. Animals were monitored daily, and tumor volumes were measured using calipers 3 times per week. Mice were euthanized when the termination criteria were met.

### Termination Criteria

Mice were euthanized when tumor volumes reached 1,000 mm^3^ (length × width × height) or other predetermined termination criteria were met (weight loss > 15% for 2 consecutive days or 20% weight loss from initial weight, serious bleeding, necrosis or ulceration of the tumor, scruffiness or lack of grooming over 5 d, lethargy over 3 d, weakness/balance issues over 5 d, hunchback appearance, diarrhea, or hypothermia).

### Statistical Analyses

Acquired data were statistically analyzed by a Student *t* test via Excel (Microsoft Corp.) and GraphPad Prism software (version 10). Acquired *P* values of less than 0.05 were considered statistically significant.

## RESULTS

### In Vitro Binding and Ex Vivo Biodistribution of [^212^Pb]Pb-DOTAM-GRPR1

**FIGURE 2.**
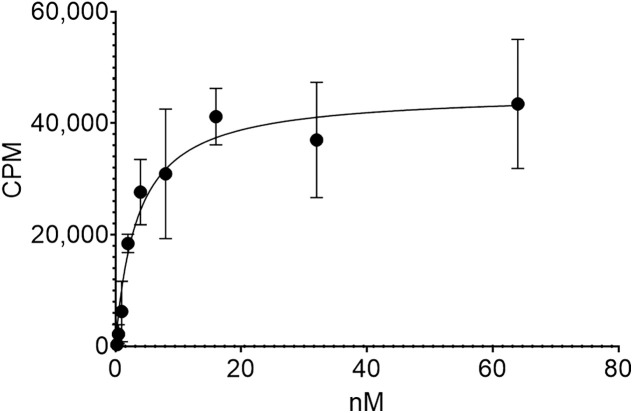
Saturation binding assay for [^212^Pb]Pb-DOTAM-GRPR1 to PC-3 cells with increasing levels of drug measured as increasing counts per minute (CPM). Average was taken of 3 wells per group and 35,000 cells per well.

Binding of [^212^Pb]Pb-DOTAM-GRPR1 in GRPR-expressing PC-3 cells was measured using an in vitro saturation binding assay (Fig. 2). A K_d_ of 3.93 ± 0.47 nM was determined, which is in line with previously reported GRPR-targeting peptides, including ^177^Lu-DOTAGA-PEG2-RM26, which has a K_d_ of 0.4 nM (24).

**FIGURE 3.**
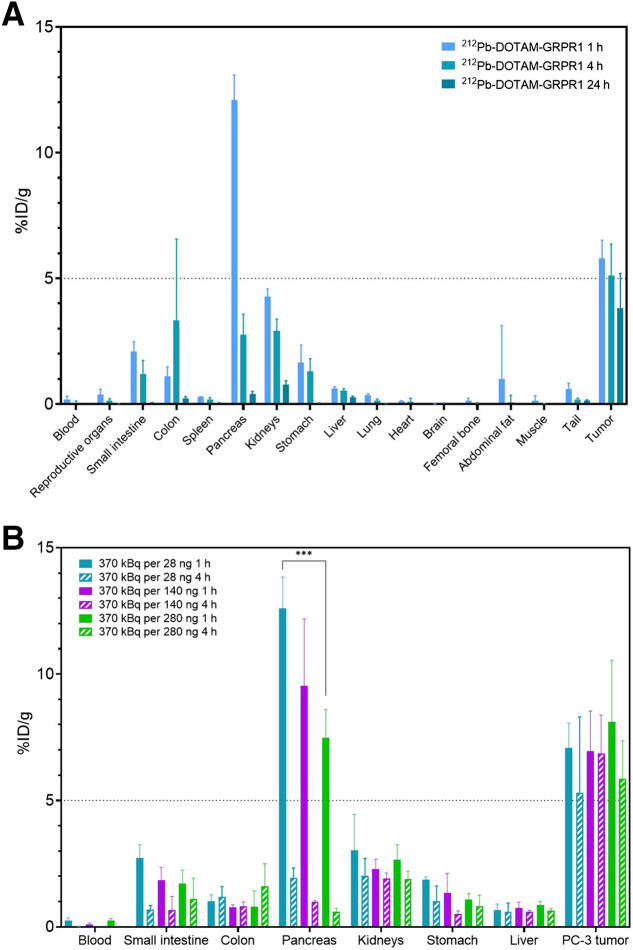
In vivo distribution of [^212^Pb]Pb-DOTAM-GRPR1. (A) In athymic nude male mice carrying subcutaneous PC-3 tumors, 370 kBq of drug were administered and organs were collected from 5 mice per time point: 1, 4, and 24 h after injection. (B) At different specific activity in PC-3 tumor-bearing mice, 370 kBq per 28, 140, or 280 ng of DOTAM-GRPR1 were administered and organs were collected from 5 mice per time point: 1, 4, and 24 h after injection. Tissue uptake is expressed as %ID/g ± SD (*n* = 5). All tissues with uptake below 2% at 1 h were excluded from graph. ***P = 0.00013 between 280 ng of pancreas and 28 ng of pancreas.

[^212^Pb]Pb-DOTAM-GRPR1 presents a safe biodistribution profile in tumor-free CD-1 mice. Analysis of the biodistribution of [^212^Pb]Pb-DOTAM-GRPR1 did not reveal any unexpected accumulation in normal organs. All tissues were well below 10 %ID/g at 4 h after drug administration. There was initial high uptake of more than 30% in the pancreas, which is likely due to the known GRPR expression in this organ. The pancreas and kidneys showed the highest nontarget uptake, but these organs also showed significantly less accumulation by 24 h after injection, whereas the average PC-3 tumor uptake was approximately 5 %ID/g at 1 h after drug administration and remained constant for 4–24 h after drug administration (Fig. 3A). Moreover, we demonstrated that the low specific activity reduced off-target accumulation. This was particularly noticeable at the level of the pancreas, in which an increase from 28 to 280 ng reduced pancreatic uptake by about 30%. However, the low specific activity did not negatively impact tumor uptake (Fig. 3B).

### Buffer Optimization to Improve Peptide Stability

**FIGURE 4.**
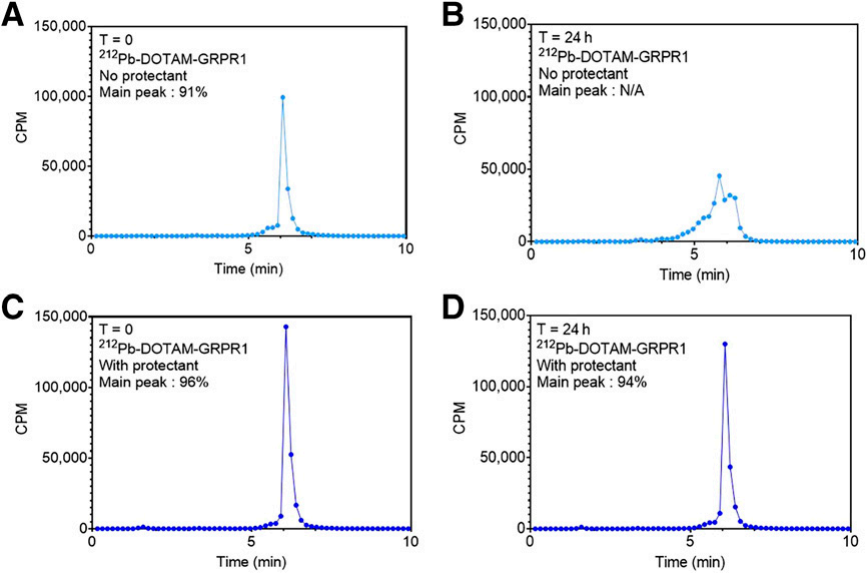
Radio–high-performance liquid chromatography showing optimized formulation conditions compared with no excipients added to chelation reaction. When chelation occurs with just ^212^Pb and peptide, oxidation is visible at time of 0 h with percent main peak of 91% (A), which becomes unquantifiable at time of 24 h (B). However, with addition of ethanol, ascorbic acid, and polysorbate 80, percent main peak is 96% at time of 0 h (C) and remains high at 94% after 24 h (D). CPM = counts per minute; N/A = not applicable; T = time.

We previously showed that the addition of ascorbic acid to the chelation reaction could minimize oxidation and improve binding of ^212^Pb-labeled peptides (9). However, additional optimization of [^212^Pb]Pb-DOTAM-GRPR1 was conducted to include ethanol and polysorbate 80 to further reduce oxidation and nonspecific adhesion, respectively. An optimized formulation of 20 mM ascorbic acid, 5% ethanol, and 0.02% polysorbate 80 minimized drug stickiness and limited oxidation (from 96% to 94% over 24 h) as measured by radio–high-performance liquid chromatography. When no excipients were added, the drug product immediately showed signs of oxidation at a time of 0 h, with a main peak of 91%. The main peak was not accurately quantifiable at 24 h (Fig. 4).

### Homogeneity and Tumor Size

**FIGURE 5.**
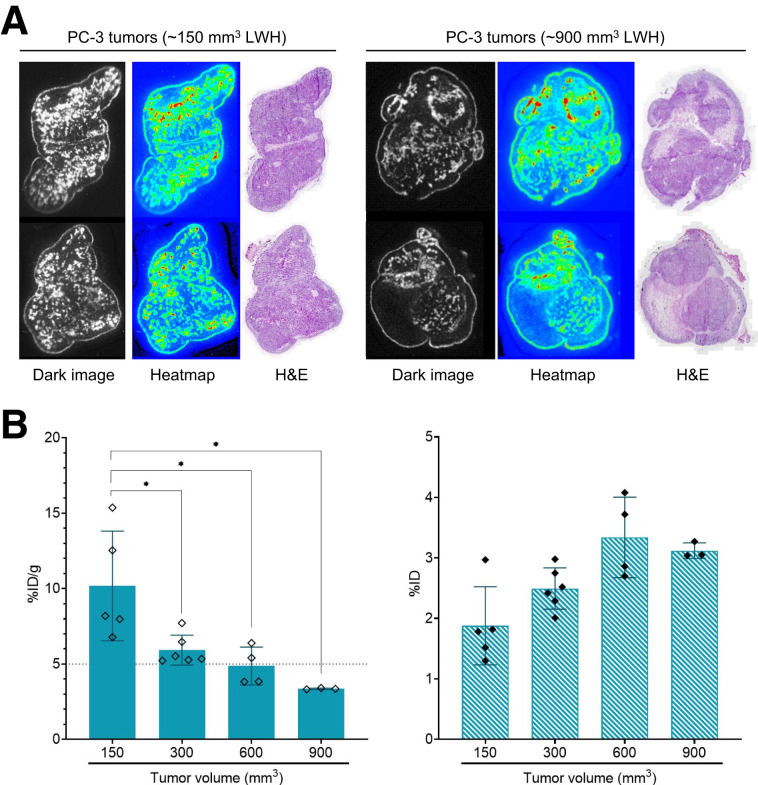
Biodistribution in varying-size tumors and drug microdistribution in tumor using α-imaging of [^212^Pb]Pb-DOTAM-GRPR1. Microdistribution of [^212^Pb]Pb-DOTAM-GRPR1 in cryosectioned PC-3 xenograft tissue samples was imaged using high-sensitivity iXon Ultra 888 EMCCD camera. (A) Left panels show sections of tumors measuring approximately 150 mm^3^ (length × width × height), and right panel shows sections of tumors measuring approximately 900 mm^3^. (B) Biodistribution of drug at 4 h after injection in tumors measuring 150 mm^3^ (*n* = 5), 300 mm^3^ (*n* = 6), 600 mm^3^ (*n* = 4), and 900 mm^3^ (*n* = 3). In left panel, tissue uptake is expressed as %ID/g ± SD (*P* < 0.05). Right panel is expressed as %ID ± SD. H&E = hematoxylin and eosin; LWH = length × width × height.

We investigated the microdistribution of [^212^Pb]Pb-DOTAM-GRPR1 in PC-3 tumors under improved peptide preparation conditions. α-imaging and hematoxylin and eosin staining of tumors treated with [^212^Pb]Pb-DOTAM-GRPR1 revealed a homogeneous distribution of the drug at various tumor sizes of up to 900 mm^3^, except for the necrotic areas (Fig. 5A). Notably, the presence of necrotic patches resulted in a negative correlation between tumor volume and tumor uptake, which was visible in larger tumors only when uptake was presented in %ID/g but not in %ID (Fig. 5B). This observation contrasts with previous findings using [^212^Pb]Pb-DOTAMTATE that indicated that tumor size did not affect tumor uptake (9).

### Dosimetry and Absorbed Dose Calculation

Murine and human-derived projections of the absorbed dose to human tissues were calculated primarily to identify dose‐limiting organs and to provide guidance regarding the amount of activity in [^212^Pb]Pb-DOTAM-GRPR1 as per title 21 of *Code of Federal Regulations,* section 361.1(b)(3)(i). α-radiation comprised most of the total absorbed dose. The linear energy transfer of α-particles is approximately 400 times greater than that of photons and β-particles. This difference in energy deposition density leads to fundamentally different biologic effects. Accordingly, the absorbed doses for these different emission types should be weighted appropriately to reflect likely biologic effects. The relative biological effectiveness (RBE) is the appropriate weighting factor for deterministic effects such as tissue toxicity and tumor kill efficacy. The tissue-absorbed doses (with RBE values of 3 and 5) for ^212^Pb compounds were calculated.

In mice, the kidneys and pancreas revealed the highest doses for both male and female animals (Table 1). On the basis of the calculated mean effective absorbed dose, the kidneys were shown to be dose-limiting. A previous study using external-beam radiotherapy found that 18–23 Gy in the whole kidney volume led to a 5% risk of kidney injury over 5 y (25). The 23-Gy threshold would be reached at 13.6 GBq of [^212^Pb]Pb-DOTAM-GRPR1 for female mice at an RBE of 3 or 8.3 GBq for female mice at an RBE of 5 based on the projected mean absorbed dose obtained by modeling (Table 1). Even at the maximum clinically relevant cumulative administered dose of 1.1 GBq of [^212^Pb]Pb-DOTAM-GRPR1, the estimated absorbed dose to the dose-limiting organ, the kidney, is approximately 7.5-fold lower than the 23-Gy threshold limit.

**TABLE 1.** Estimated Absorbed Doses at Clinically Relevant and Maximum Administered Activities of [^212^Pb]Pb-DOTAM-GRPR1 for Female Mice

	Clinically relevant			Maximum	Dose limit
Site	0.111 GBq (3 mCi)	0.555 GBq (15 mCi)	1.110 GBq (30 mCi)	8.29 GBq (224.05 mCi)	
Kidneys	0.308	1.540	3.080	23.00	23
Pancreas	0.287	1.434	2.867	21.42	
Liver	0.109	0.543	1.086	8.11	30
Stomach (stem cell layer)	0.044	0.219	0.438	3.27	45
Lungs (alveolar–interstitial)	0.022	0.108	0.216	1.61	65
Urinary bladder wall	0.021	0.103	0.206	1.54	20
Spleen	0.018	0.091	0.183	1.37	26
Heart wall	0.007	0.036	0.071	0.53	45
Red marrow	0.001	0.004	0.008	0.06	2

Absorbed doses are in grays (RBE, 5).

### ^212^Pb-DOTAM-GRPR1 Dose and Toxicity Studies

We performed a dose escalation study in tumor-free nude male mice to assess the toxicity profile of [^212^Pb]Pb-DOTAM-GRPR1 and determine the safe dose range for subsequent efficacy studies. As shown in Supplemental Figure 1 (supplemental materials are available at http://jnm.snmjournals.org), even at the highest dose (a single 1,665-kBq dose or 3 fractionated doses of 555 kBq each), body weight loss was minimal after administration and was followed by body weight gain, which steadily increased throughout the study.

Histopathologic examinations revealed that all groups were essentially within the normal limits. [^212^Pb]Pb-DOTAM-GRPR1 was well tolerated at the doses used, with no signs of radiation or lead injury. The kidneys, gastrointestinal mucosa, and bone marrow were unremarkable, as were the other organs. The toxic dose was therefore determined to be more than 1,665 kBq in the male athymic nude mouse strain.

### Efficacy Studies Using ^212^Pb-DOTAM-GRPR1

**FIGURE 6.**
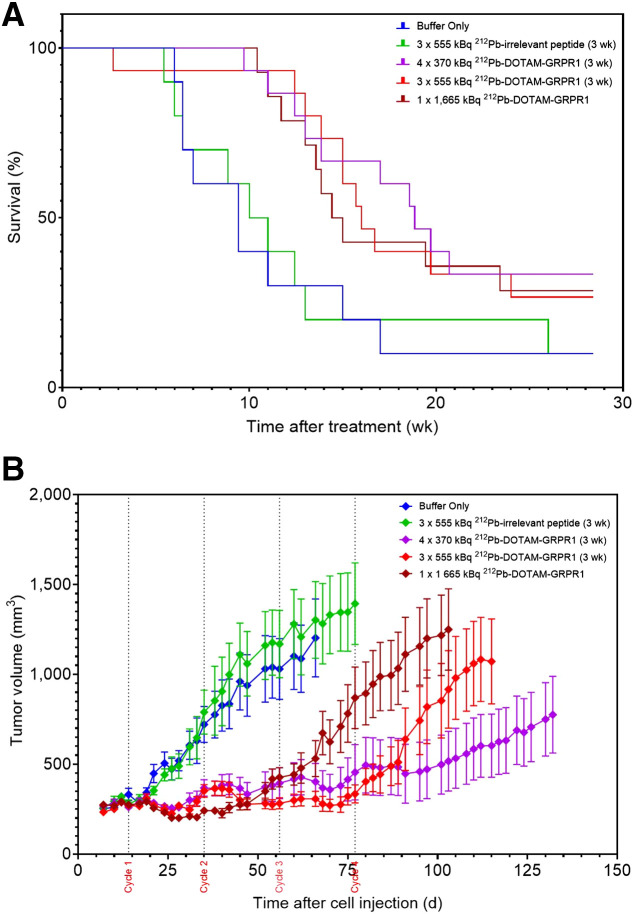
Percentage animal survival (A) and average tumor development (B) after 1–4 cycles of [^212^Pb]Pb-DOTAM-GRPR1 in subcutaneous PC-3 model. Shown are data for no treatment (*n* = 10), 1 × 1,665 kBq of [^212^Pb]Pb-DOTAM-GRPR1 (*n* = 15); 3 × 555 kBq of [^212^Pb]Pb-DOTAM-GRPR1 (*n* = 15), 4 × 370 kBq of [^212^Pb]Pb-DOTAM-GRPR1 (*n* = 15), and 3 × 555 kBq of [^212^Pb]Pb-irrelevant peptide (*n* = 10). Tumor volume is expressed as mm^3^ ± SEM.

One to 4 intravenous administrations of [^212^Pb]Pb-DOTAM-GRPR1 (Figs. 6A and 6B; Table 2) improved the survival of PC-3 tumor–bearing mice compared with the control groups. At 15 wk after cell injection, whereas only 10% of the control group mice were still alive, 33.3% of mice treated with 4 doses of 370 kBq, 26.7% of those treated with 3 doses of 555 kBq, and 28.6% of those treated with a single dose of 1,665 kBq were still alive. The median survival times of the control group mice were approximately 9 and 10 wk (for buffer only and 3 × 555 kBq ^212^Pb-irrelevant peptide controls, respectively); however, mice administered a single injection of 1,665 kBq of [^212^Pb]Pb-DOTAM-GRPR1 had a survival time of 14 wk, and mice that received four 370-kBq doses at 3-wk intervals had survival times of up to 19 wk.

**TABLE 2.** Efficacy of ^212^Pb-DOTAM-GRPR1 Treatment on PC-3 Tumors

Group	Time to median survival (wk)	Percentage survival at study termination
Buffer only	9.4	10%
3 × 555 kBq of ^212^Pb-irrelevant peptide	10.5	10%
4 × 370 kBq of ^212^Pb-DOTAM-GRPR1	18.9	33%
3 × 555 kBq of ^212^Pb-DOTAM-GRPR1	16	27%
1 × 1,665 kBq of ^212^Pb-DOTAM-GRPR1	14.7	29%

## DISCUSSION

GRPR is an attractive therapeutic target for GRPR-expressing cancers, such as metastatic castration-resistant prostate cancer, breast, colorectal, cervical, and cutaneous melanoma cancers. In this preclinical study, we extensively demonstrated the feasibility, safety, and therapeutic potential of [^212^Pb]Pb-DOTAM-GRPR1 as a treatment for GRPR-positive tumors.

Our in vitro results showed binding to the target receptor with a reasonable K_d_ and that the optimized chelation formulation increased the stability of the drug well beyond 24 h. Nonclinical results showed that [^212^Pb]Pb-DOTAM-GRPR1 presented a safe biodistribution profile with a considerably low activity in nontumor tissues. The kidneys and pancreas received the highest dose; however, faster washout was observed from the pancreas than from tumor tissues. These observations are consistent with the pharmacokinetic data of JVM4168 (26), which is a DOTA-conjugated peptide that shares the same binding motif as DOTAM-GRPR1. Excretion analysis indicated a predominantly renal route of excretion. As kidney protection agents are often given with targeted radiotherapies to minimize nephrotoxicity, the renal blocking effect of positively charged amino acids or colchicine was investigated in vivo, but neither agent reduced the kidney retention of ^212^Pb-radiolabeled DOTAM-GRPR1; therefore, their use in clinical trials is not warranted. It is worth noting that [^212^Pb]Pb-DOTAM-GRPR1 uptake in the kidney even in the absence of renal blocker remains well below kidney retention values of radiolabeled somatostatin receptors ligands, such as [^212^Pb]Pb-DOTAMTATE, in the presence of a renal blocker (9). In fact, even at the planned starting dose of about 111 MBq in the clinical study, the estimated absorbed dose to the kidneys (dose-limiting tissue) was about 75-fold lower than the 23-Gy threshold toxicity limit (27). Additionally, we demonstrated that lowering the specific activity further reduced off-target accumulation, especially in the pancreas, but did not negatively impact tumor uptake. These observations are in line with published results using other radiolabeled GRPR antagonist peptides (26,28) and could be explained by differences in the vasculature (healthy tissue vs. diseased) and metabolic degradation.

It was previously demonstrated that, as targeting agents, GRPR antagonists are superior to GRPR agonists because of the former’s higher number of binding sites, higher metabolic stability, or stronger interaction with the receptor, which explains the continuous tumor retention over time (29). Consistently, in the pilot C-BOBCAT trial, which examined [^64^Cu]Cu-SAR-bombesin (a comparable GRPR antagonist peptide), PET imaging in female patients with recurrent metastatic estrogen receptor–positive/progesterone receptor–positive/human epidermal growth factor receptor 2–negative breast cancer revealed initial uptake of the peptide by the pancreas with a rapid washout between 4 and 24 h, whereas high and strong retention in tumor lesions was visualized up to at least 24 h (30).

Using α-camera imaging, we observed a homogeneous microdistribution of [^212^Pb]Pb-DOTAM-GRPR1 in PC-3 tumors. Areas of low compound imaging, corresponding to areas of necrosis in the tissue, were especially visible in large tumors (>600 mm^3^ [length × width × height]) and correlated with lower tumor uptake in large tumors (%ID/g but not %ID).

An acute toxicity study was conducted to determine the appropriate dose of [^212^Pb]Pb-DOTAM-GRPR1 in athymic nude male mice. The results revealed that all animals survived for the full 30-d period after receiving 1,665 kBq of [^212^Pb]Pb-DOTAM-GRPR1 with no hematologic toxicity or weight loss. In contrast, 100% of animals injected with 1,480 kBq of [^212^Pb]Pb-DOTAMTATE died by 8 d after injection (9). Since [^212^Pb]Pb-DOTAMTATE presented a favorable safety profile during a phase 1 clinical trial (12,13), [^212^Pb]Pb-DOTAM-GRPR1 would be expected to exhibit an even greater level of safety.

Administration of [^212^Pb]Pb-DOTAM-GRPR1 to mice bearing GRPR-positive PC-3 xenografts revealed the targeted α-therapy to have high antitumor efficacy. Under optimized conditions, median survival was approximately 9–10 wk for the control mice but 14–19 wk for [^212^Pb]Pb-DOTAM-GRPR1–treated animals. Similarly, animals treated with [^177^Lu]Lu-JMV4168 presented a median survival of 79 d (11.2 wk), whereas control groups showed a median survival of 35 d (5 wk) (31). Nevertheless, the treated animals showed regrowth of tumors approximately 32 ± 4 d after [^177^Lu]Lu-JMV4168 regardless of the treatment dose, whereas about 30% of the animals were tumor-free 28 wk after ^212^Pb-DOTAM-GRPR1 administration irrespective of the dosage.

## CONCLUSION

Taken together, the combined findings from our preclinical studies demonstrate the safety, tolerability, and efficacy of [^212^Pb]Pb-DOTAM-GRPR1 and suggest beneficial clinical implications for the treatment of GRPR-positive tumors with this targeted α-therapy. Thus, further exploration of [^212^Pb]Pb-DOTAM-GRPR1 in clinical trials is warranted.

## DISCLOSURE

No potential conflict of interest relevant to this article was reported.

## Notes

KEY POINTS **QUESTION:** What are the preclinical properties of a ^212^Pb-radiolabeled GRPR-targeting peptide, and does it have potential for clinical application? **PERTINENT FINDINGS:** [^212^Pb]Pb-DOTAM-GRPR1 presents a safe biodistribution profile, has a toxic dose estimated to be over 1,665 kBq, and can substantially increase median survival. **IMPLICATIONS FOR PATIENT CARE:** Our results suggest that targeted α-therapy using [^212^Pb]Pb-DOTAM-GRPR1 could have beneficial clinical implications for the treatment of GRPR-positive tumors.
